# Social epidemiology of the Mediterranean-dietary approaches to stop hypertension intervention for neurodegenerative delay (MIND) diet among early adolescents: the Adolescent Brain Cognitive Development Study

**DOI:** 10.1038/s41390-023-02959-7

**Published:** 2023-12-15

**Authors:** Jason M. Nagata, Ammal Bashir, Shayna Weinstein, Abubakr A. A. Al-Shoaibi, Iris Yuefan Shao, Kyle T. Ganson, Alexander Testa, Andrea K. Garber

**Affiliations:** 1 Department of Pediatrics, University of California, San Francisco, San Francisco, CA USA; 2 Factor-Inwentash Faculty of Social Work, University of Toronto, Toronto, ON Canada; 3 Department of Management, Policy and Community Health, University of Texas Health Science Center at Houston, Houston, TX USA

## Abstract

**Background:**

The purpose of our study was to understand the relationship between sociodemographic factors and adherence to the MIND (Mediterranean-DASH [Dietary Approaches to Stop Hypertension] Intervention for Neurodegenerative Delay) diet in a demographically diverse national population-based sample of 9–12-year-olds in the US.

**Methods:**

We analyzed data from the Adolescent Brain and Cognitive Development (ABCD) Study (Year 1, *N* = 8333). Multivariable linear regression analysis was used to identify associations between MIND diet score and sociodemographic factors, including race/ethnicity, household income, parent education level, age, sex, and sexual minority status.

**Results:**

Compared to White adolescents, Latino adolescents showed the greatest adherence to the MIND diet. Boys had lower adherence to the MIND diet than girls. Lower household income was associated with lower adherence to the MIND diet. Older age was associated with lower adherence to the MIND diet. Sexual minorities had a lower adherence to the MIND diet when compared to their heterosexual counterparts.

**Discussion:**

Female sex, Latino ethnicity, Asian and Black race, high household income, heterosexual sexual orientation, and younger age were associated with higher adherence to the MIND diet. These sociodemographic differences can inform targeted screening and counseling for clinicians and public health organizations among diverse adolescent populations.

**Impact Statement:**

- Sociodemographic disparities in diet quality have been documented, but none have explored adherence to the MIND (Mediterranean-DASH [Dietary Approaches to Stop Hypertension] Intervention for Neurodegenerative Delay) diet in early adolescence.
- In this demographically diverse sample of 9–12-year-old early adolescents in the U.S., we found notable and nuanced sociodemographic disparities in adherence to the MIND diet.
- Sociodemographic factors associated with higher adherence to the MIND diet included female sex, Latino ethnicity, high household income, heterosexual sexual orientation, and younger age.

## Introduction

Adolescence is a crucial period of immense growth and development, a time when nutrient requirements are the highest in the entire lifespan.^1,2^ Diet quality during adolescence can also shape lifelong food preferences,^3^ and can play a role in preventing several chronic diseases, such as cardiovascular disease, and depression.^4–6^ The USDA Dietary Guidelines for children and adolescents outline a nutritious and adequate diet quality for children and adolescents.^7^ These guidelines align with the structure created by Mediterranean-DASH (Dietary Approaches to Stop Hypertension) Intervention for Neurodegenerative Delay (MIND) diet. Both diets emphasize core food groups of vegetables, fruits, whole grains, proteins such as seafood, poultry, and nuts and seeds.^7,8^ Adolescents’ poor adherence to the recommended dietary guidelines has been well-documented over time.^9,10^ Measured by the 2015 Healthy Eating Index (HEI-2015), adolescents aged 9–13 (HEI = 52) and 14–18 (HEI = 49) had lower diet quality scores than the American population’s average total (HEI = 58) in 2017–2018.^11^

The MIND diet shares many food groups from both the Mediterranean and DASH diets and emphasizes plant-based options.^8^ The DASH diet was clinically designed to lower blood pressure, whereas the Mediterranean diet prescribes a rich diet quality (fruits, vegetables, nuts, seeds, legumes, potatoes, whole grains, seafood, and olive oil) that has been extensively studied and shown to reduce mortality, cardiovascular incidence, cancer, and neurodegenerative diseases among adults.^12,13^ Overall, among youth, adhering to the Mediterranean-style diet is linked to improved cognition and academic achievement^14^ and may decrease the risk of chronic non-communicable diseases later in life.^15^ Additionally, this diet has been shown to lower the risk of depression in adolescents,^16^ has been associated with fewer ADHD diagnoses among children,^17^ and decreased the cardiovascular risk of obese youth by lowering BMI, glucose, and lipid profiles.^18^ There have been limited clinical trials and cross-sectional studies of adherence to the DASH diet among adolescents. Among children who were overweight, following the DASH diet was associated with a lower risk of obesity.^19^ For youth with hemophilia, higher adherence to the DASH diet had beneficial effects on blood pressure and lipid profiles.^20^ Adolescents with elevated blood pressure and hypertension also showed effective improvements in initial systolic blood pressure, longer-term endothelial function, and diet quality when following the DASH diet instead of routine care.^21^ Additionally, the DASH diet was protective against gastroesophageal reflux disease.^22^ In Brazil, among a large adolescent sample (*n* = 71,553), no association was found between arterial hypertension or overweight/obesity and the DASH diet.^23,24^

The MIND diet was designed for aging populations to improve brain health and is associated with a lower risk of all-cause mortality over 12 years,^25^ slower cognitive decline,^8^ decreased severity of anxiety disorders,^26^ and reduced risk of Alzheimer’s disease^27^ and Parkinson’s disease.^28^ Adolescent brain development has been labeled as a period of vulnerability and opportunity,^29^ during which the brain undergoes fundamental reorganization,^30^ and important maturation. During this period, a focus on brain, behavior, and social-context interactions is important.^29^ Nutrition plays a crucial role in neurogeneration, axonal and dendritic growth, synaptic formation, and the myelination of axons.^31^ Only one study from Tehran, Iran has investigated the MIND diet related to children with a smaller and non-representative sample, and showed a lower risk of obesity in children aged 6 years old who adhered to the diet.^32^

Social epidemiology focuses on the intersection of social and structural factors that influence health.^33,34^ For instance, demographic (e.g., sex, race/ethnicity, and sexual orientation) and socioeconomic (e.g., income and education) factors could be associated with an individual’s health outcomes.^33,34^ Although sociodemographic factors have not been investigated with the MIND diet, prior literature has highlighted that diet quality scores were significantly lower for non-Hispanic black children compared with children of other races and higher for Mexican-American children compared to non-Hispanic White children.^35,36^ There were also persistent differences in household income and household food security status, but mixed results for parental education level.^9,10^

Given the gaps in the literature, the objective of our study was to describe participants’ adherence to the 14 categories of the MIND diet among a socio-demographically diverse, population-based sample of 9 to 12-year-old early adolescents in the United States. Our second aim was to explore differences in adherence to the MIND diet by race/ethnicity, sex, sexual orientation, and socioeconomic status.

## Methods

### Study population

We analyzed data from the Adolescent Brain Cognitive Development (ABCD) study (Year 1). The ABCD study is a longitudinal study of brain development, cognitive growth, and adolescent health that began in 2016. It involves the recruitment of 11,875 children from 21 sites across the United States. During the Year 1 visit, children were between the ages of 9 to 12. Exclusion criteria included participants who had missing data for the Child Nutritional Assessment, race/ethnicity, sex, sexual minority status, household income, or parental education level (*n* = 2892), leaving 8,333 participants included in the analysis (Appendix A). Institutional review board (IRB) approval was obtained from the University of California, San Diego (IRB approval number 160091) as well as each respective study site. Informed written consent was obtained from caregivers, and each child participant gave written assent.

### Dependent variables

#### Child nutrition assessment

Parents completed a Child Nutrition Assessment (CNA) in Year 1, which was a derivate of the MIND (Mediterranean-DASH intervention for neurodegenerative delay) diet questionnaire. The MIND diet questionnaire is a 15-item questionnaire that assesses intake frequencies for whole grains, vegetables, leafy vegetables, berries, nuts, poultry, beans, fish, wine, and olive oil. It also asks about the intake of foods that have been associated with higher risk of neurodegeneration, such as fast and fried foods, pastries and sweets, butter, and cheese. The assessment given by the ABCD study omitted the category of wine, leaving 14 categories of food (Appendix B). The CNA asks whether the child consumed a specific serving size of each food group weekly, over the last year. A “yes” answer was coded as a 1, while a “no” answer was coded as a 0. For each participant survey, the total score was calculated, and this sum score was used as our dependent variable and measure of overall nutritional status. Given that adolescents tend to underreport dietary energy intake and are prone to errors when completing food records,^37,38^ the nutritional status data was reported by parents who have been shown to be valid reporters for their children’s diet.^39^

#### Independent variables

Parents were asked to report sociodemographic characteristics of their children, including participants’ sex at birth (male or female), race/ethnicity (Asian, Black, Latino, Native American, White, or other), annual household income (less than $25 K, $25K-$50 K, $50K-$75 K, $75K-$100 K, $100K-$200 K, or greater than $200 K), highest parent education level (college education or more, or high school education or less), and age. Sexual orientation was reported by the adolescent participants (“Are you gay or bisexual?”; yes, maybe, no, don’t understand the question, decline to answer).

#### Anthropometrics

Height (Carpenter’s square, steel tape measure) and weight (Health-o-meter 844KL High-Capacity Digital Bathroom Scale; Jarden Corporation; Rye, NY) were assessed with two-to-three measurements by a trained research assistant at each site. Body Mass Index (BMI) was computed using the standard formula, weight (kilograms) divided by height (meters) squared (BMI = weight/height^2^). Height, weight, and BMI were converted into sex and age-specific z-scores in accordance with CDC growth curves.^40,41^

#### Statistical analysis

Data analyses for this study were performed in 2023 using Stata 18. Descriptive statistics including mean, standard deviation, and percentages were calculated. Multivariable linear regression analysis was conducted to investigate the associations between sociodemographic factors (age, sex, race/ethnicity, sexual orientation, household income, parents’ highest education) and MIND adherence measured via the total nutrition score, adjusting for study site. We additionally conducted sensitivity analyses adjusting for BMI-for-age z-score, height-for-age z-score, and weight-for-age z-score. Propensity weights were applied to match key sociodemographic variables in the ABCD Study to the American Community Survey from the US Census.^42^

**Table 1:** Sociodemographic and nutrition characteristics of Adolescent Brain Cognitive Development (ABCD) Study participants (*N* = 8333).

Sociodemographic characteristics (baseline)	Mean (SD) / %
Age (years)	10.90 (0.64)
Sex (%)	
Female	48.9%
Male	51.0%
Race/ethnicity (%)	
White	56.7%
Latino / Hispanic	19.3%
Black	14.4%
Asian	5.3%
Native American	2.9%
Other	1.3%
Sexual minority (%)	
No	87.4%
Yes	4.5%
Maybe	3.9%
Don’t understand the question	3.1%
Decline to answer	1.1%
Household income (%)	
$24,999 or less	15.4%
$25,000 to $49,999	18.1%
$50,000 to $74,999	15.7%
$75,000 to $99,999	14.7%
$100,000 to $199,999	26.8%
$200,000 and greater	9.4%
Parent’s highest education	
College education or more	89.6%
High school education or less	10.4%
Anthropometric factors	
BMI-for-age z-score	0.48 (1.54)
Height-for-age z-score	0.33 (1.13)
Weight-for-age z-score	0.05 (1.18)
MIND diet sum score	8.05 (2.40)
Individual MIND diet items (% responding yes)	
Whole grains ≥3 times a day	62.3%
Green leafy vegetables ≥6 times a week	43.6%
Other vegetables ≥1 times a day	82.5%
Berries ≥2 times a week	64.1%
Red meat and meat products <4 times a week	75.0%
Fish ≥1 times a week	36.0%
Poultry ≥2 times a week	87.3%
Beans ≥4 times a week	26.7%
Nuts ≥5 times a week	23.1%
Fast or Fried food <1 times a week	65.8%
Olive oil used as the primary oil	66.0%
Butter or Margaine <1 Tablespoon a day	78.0%
Cheese <1 times a week	30.4%
Pastries or Sweets <5 times a week	59.8%

ABCD propensity weights were applied based on the American Community Survey from the US Census.

*SD* standard deviation, *MIND* Mediterranean-DASH [Dietary Approaches to Stop Hypertension] Intervention for Neurodegenerative Delay.

## Results

### Table 1. Sociodemographic characteristics

Table 1 describes the sociodemographic characteristics of the (*N* = 8333) participants included and the percentage of participants responding “yes” to each of the questions on the CNA. The sample was split evenly according to sex, and the average age of participants was 10.9 years. The sample was also racially diverse (43.3% racial minority). Consuming nuts more than five times a week was the category least frequently followed by participants. The average nutrition sum score was 8.08 points out of 14.

**Table 2:** Sociodemographic associations with MIND diet sum score in the Adolescent Brain Cognitive Development (ABCD) Study.

Independent variables	MIND diet sum score	
	Coefficient (95% CI)	p-value
Age	**-0.16 (-0.25, -0.06)**	**0.001**
Sex		
Female	Reference	
Male	**-0.2 (-0.32, -0.08)**	**0.001**
Race/ethnicity		
White	Reference	
Asian	**0.37 (0.05, 0.68)**	**0.02**
Black	**0.27 (0.05, 0.48)**	**0.01**
Latino / Hispanic	**0.66 (0.45, 0.87)**	**<0.001**
Native American	0.26 (-0.09, 0.62)	0.14
Other	0.51 (-0.13, 1.10)	0.12
Sexual minority		
No	Reference	
Yes	**-0.67 (-0.97, -0.37)**	**<0.001**
Maybe	-0.29 (-0.61, 0.01)	0.06
Don’t understand the question	-0.11 (-0.44, 0.23)	0.53
Decline to answer	-0.37 (-0.97, 0.23)	0.22
Household income		
$24,999 or less	-0.13 (-0.39, 0.14)	0.36
$25,000 to $49,999	**-0.50 (-0.73, -0.26)**	**<0.001**
$50,000 to $74,999	**-0.46 (-0.67, -0.24)**	**<0.001**
$75,000 to $99,999	**-0.39 (-0.58, -0.19)**	**<0.001**
$100,000 to $199,999	**-0.23 (-0.39, -0.06)**	**<0.001**
$200,000 and greater	Reference	
Parent’s highest education		
College education or more	Reference	
High school education or less	-0.11 (-0.38, 0.16)	0.43

Bold indicates *p* < 0.05. Models represent the abbreviated output from a linear regression model including adjustment for age, sex, race/ethnicity, sexual orientation, household income, parent education, data collection period, and study site. Propensity weights from the Adolescent Brain Cognitive Development Study were applied based on the American Community Survey from the US Census.

*MIND* Mediterranean-DASH (Dietary Approaches to Stop Hypertension) Intervention for Neurodegenerative Delay.

### Table 2. Associations of sociodemographic factors with nutrition sum score

Table 2 presents the adjusted linear regression models that analyze the association between sociodemographic factors and the nutrition sum score. Boys had lower nutrition scores than girls by 0.20 points (95% confidence interval [CI] −0.32, −0.08). Additionally, sexual minorities had a lower nutrition score (−0.64, 95% CI −0.97, −0.37) compared to their heterosexual peers. Latino/Hispanic (0.66, 95% CI 0.45, 0.87), Black (0.27, 95% CI 0.05, 0.48), and Asian (0.37, 95% CI 0.05, 0.68) participants had a higher nutrition score when compared to the White participants.

### Sensitivity analyses

Sensitivity analyses of linear regression models analyzing the association between sociodemographic factors and the nutrition sum score adjusting for BMI-for-age z-score, height-for-age z-score, and weight-for-age z-score were mostly similar (Appendix C). Higher BMI-for-age z-score and weight-for-age z-score were associated with a lower nutrition sum score (Appendix D).

## Discussion

Using the MIND diet questionnaire in a diverse sample of early adolescents in the United States, we found that on average early adolescents met 8 out of 14 components of the MIND diet score. This is similar to a previously reported average score of 7.4 (range: 2.5–12.5) out of 15 [which included the wine consumption category] in a sample of elderly adults, for whom this diet was designed.^8^ Adolescents had the highest adherence to meeting the MIND diet recommendations of eating poultry and vegetables and using olive oil as the primary cooking oil. Sociodemographic factors associated with higher adherence to the MIND diet included female sex, Latino ethnicity, Asian and Black race, high household income, heterosexual sexual orientation, and younger age.

### Race/ethnicity

Latino/Hispanic, Asian, and Black early adolescents had better adherence to the MIND diet when compared to White adolescents, respectively. This is consistent with previous research showing that diet quality scores were higher for Mexican-American children than non-Hispanic white children.^35,36^ Despite immigrant status and decreased health resources, the Latino Paradox describes the phenomenon whereby Latino immigrants have better overall health outcomes than their US-born counterparts.^43^ While previous studies have looked at this paradox in Mexican-American adults, we were able to hone in on how early adolescents rank compared to other racial/ethnic minorities in adhering to the MIND diet. A 2022 study assessed how adherence to the Mediterranean diet in Latino/Hispanic identifying adults affected cognition, which found that adherence is high in recent immigrants and decreases with more time spent in the United States.^44^ Recent migrants have been shown to have certain positive nutritional health outcomes, while acculturation leads to decreasing health outcomes over time,^45^ which could partially explain the higher scores among Latino, Asian, and Black adolescents.

It was interesting to find that Black adolescents showed higher adherence to the MIND diet than White adolescents. There is a study among youth that showed Black and Asian youth had significantly higher intakes of vegetables while White youth had the lowest intakes of fruits.^46^; however, other literature documents that Black children and adolescents have the poorest dietary quality when compared to other racial/ethnic groups.^35,36,47^ Black adults also have worse overall diet quality scores overall.^48–50^; however, it is worth noting that White adults showed a significant decrease in diet quality scores from 2011 to 2018 that other racial/ethnic groups did not demonstrate.^48^ Future research could confirm these findings and investigate why minority adolescent populations may report better adherence to the MIND diet.

### Household income

Adolescents who had parents with a lower annual income had worse adherence to the MIND diet when compared to those who had a high annual income (≥$200 K). There is extensive evidence that shows the association between decreased nutritional status and lower annual income. Among youth with household incomes less than 1.30 times the poverty level, there was a higher estimated proportion of youth with poor diet quality.^51^ This association may be explained by the decrease in purchasing power for fresh produce, proteins, and nuts that are critical in the MIND diet, as previous studies have shown that economic constraints can influence healthy eating and shopping preferences.^52^

### Sex

We found sex differences in nutrition scores, with males having a lower MIND diet component score when compared to their female counterparts. In general, research has shown that women have better-quality diets than men.^53^ In a sample of Swedish adolescents, girls had higher scores for healthy eating and diet diversity.^54^ However, in a sample of American adolescents, there were no apparent differences in total mean HEI scores between boys and girls.^35^ Future research should further investigate the sex differences in diet quality.

### Age

Younger age among early adolescents was associated with a higher MIND diet score. This is consistent with the literature documenting an inverse relationship between child age and diet quality.^9,10,35,47^ The tendency for older adolescents to have worse diet quality may be a result of increased marketing to this age group, autonomy, and availability in the selection of unhealthy foods.^10^ We were able to contribute to the existing body of research that confirms this trend but with another measure of nutritional adherence specific to cognitive health.

### Sexual minority status

To our knowledge, our study is the first to examine adherence to the MIND diet among sexual minority adolescents. Sexual minority adolescents had lower adherence to the MIND diet, which contrasts with prior studies examining sexual orientation and nutrition outcomes in adults. Among a cohort of adult female nurses, lesbian women had higher DASH scores than heterosexual women who had no same-sex partners.^55^; it is notable that female nurses are a cohort of women with high health literacy, which may influence their nutrition. A separate study found that gay/bisexual males had significantly higher total Healthy Eating Index [HEI-2015] scores than heterosexual males; however, lesbian/bisexual females did not differ in total or component scores from heterosexual females.^56^ An additional study of participants aged 10–23 found that gay males and “mostly heterosexual” females had higher diet quality scores than their entirely heterosexual counterparts.^57^ Possible mechanisms that could explain these findings include the discrimination, stigma, and bullying that sexual minority adolescents face, which has been associated with depression^58^ and disordered eating^59^ and subsequent diet quality. Given sexual minority status is associated with a higher risk of adverse health outcomes, such as diabetes and cardiovascular disease, it is important to also assess factors contributing to possible cognitive decline.^60^

### Limitations and strengths

Several limitations should be noted. Due to the observational nature of the study, the findings do not reflect causality. Our study observed relatively weak effect sizes; however, the negative direction of the coefficients supports the associations found. It is important to recognize that even very small associations can hold significance at the population level, and our study leverages a large, representative national sample. Slight differences on the point scale of the MIND diet score may potentially translate into notable health disparities as adolescents age. It is also well known that there are limited valid tools that exist for measuring dietary intake among children and adolescents.^61^ The MIND diet questionnaire, based on parent report, is subject to responder, recall, and social desirability bias. However, our study provides nutritional information on a large, demographically diverse population of adolescents with assessment from the parents, who are found to be valid sources of nutrition information as proxies for adolescents.^62^ A recent systematic review found that the most accurate dietary assessment of children and adolescents (aged 4–14 years) was a method that utilized the parent as the reporter.^63^ The self-reporting of adolescents poses numerous difficulties since the same behavioral factors and eating habits that influence their food choices can also influence how they report their dietary intake.^3^

### Clinical implications

Our results support the need for continued efforts from governments, the industry, clinicians, and schools to improve the diet quality of adolescents and promote brain-healthy foods during this crucial time of development. We found notable disparities in diet quality among low-income, sexual minority, and older adolescents, necessitating targeted interventions for each. The MIND diet combines the Mediterranean and DASH diets, simplifying them into ten brain-healthy food groups to consume and five unhealthy food groups to avoid.^8^ This diet also promotes heart health—aligning with #1 of the American Heart Association’s “Eat Better” principle, emphasizing fruits, vegetables, lean protein, nuts, seeds, and cooking with non-tropical oils.^64^ Educators can incorporate the MIND diet into school health curricula to establish healthy behaviors early on.^65^ Healthcare providers can address disparities among vulnerable adolescents, including those from low-income contexts and sexual minorities, through nutrition assessment,^66^ nutrition counseling,^67^ and screening for food insecurity.^68^ Clinicians and public health practitioners can advocate for “Food Is Medicine” Interventions to expand access to healthy foods in the healthcare system by producing prescriptions for adolescents.^69^ Parents, serving as role models, can also play a critical role in the development of adolescents’ eating behaviors by following the MIND diet themselves and incorporating education on healthy eating at home.^70^

### Supplementary information

Appendix

## Data Availability

Data used in the preparation of this article were obtained from the ABCD Study (https://abcdstudy.org), held in the NIMH Data Archive (NDA). Investigators can apply for data access through the NDA (https://nda.nih.gov/).

## References

[1] Corkins MR (2016). Nutrition in children and adolescents. Med. Clin. North Am..

[2] Jacob JA, Nair MKC (2012). Protein and micronutrient supplementation in complementing pubertal growth. Indian J. Pediatr..

[3] Scaglioni S (2018). Factors influencing children’s eating behaviours. Nutrients.

[4] Liese AD (2015). The dietary patterns methods project: synthesis of findings across cohorts and relevance to dietary guidance1234. J. Nutr..

[5] Dahm CC (2016). Adolescent diet quality and cardiovascular disease risk factors and incident cardiovascular disease in middle‐aged women. J. Am. Heart Assoc. Cardiovasc. Cerebrovasc. Dis..

[6] Chopra C, Mandalika S, Kinger N (2021). Does diet play a role in the prevention and management of depression among adolescents? A narrative review. Nutr. Health.

[7] U.S. Department of Agriculture. *Dietary Guidelines for Americans, 2020–2025*. U.S. Department of Health and Human Services (2020).

[8] Morris MC (2015). MIND diet slows cognitive decline with aging. Alzheimers Dement J. Alzheimers Assoc..

[9] Banfield EC, Liu Y, Davis JS, Chang S, Frazier-Wood AC (2016). Poor adherence to US dietary guidelines for children and adolescents in the national health and nutrition examination survey population. J. Acad. Nutr. Diet..

[10] Liu J, Rehm CD, Onopa J, Mozaffarian D (2020). Trends in diet quality among youth in the United States, 1999-2016. JAMA.

[11] Food and Nutrition Service. HEI Scores for Americans | Food and Nutrition Service. Accessed June 7, (2023). https://www.fns.usda.gov/hei-scores-americans.

[12] Siervo M (2015). Effects of the Dietary Approach to Stop Hypertension (DASH) diet on cardiovascular risk factors: a systematic review and meta-analysis. Br. J. Nutr..

[13] Sofi F, Abbate R, Gensini GF, Casini A (2010). Accruing evidence on benefits of adherence to the Mediterranean diet on health: an updated systematic review and meta-analysis. Am. J. Clin. Nutr..

[14] Naveed S, Lakka T, Haapala EA (2020). An overview on the associations between health behaviors and brain health in children and adolescents with special reference to diet quality. Int J. Environ. Res. Public Health.

[15] Iaccarino Idelson P, Scalfi L, Valerio G (2017). Adherence to the Mediterranean Diet in children and adolescents: a systematic review. Nutr. Metab. Cardiovasc. Dis..

[16] Zielińska M, Łuszczki E, Michońska I, Dereń K (2022). The Mediterranean Diet and the Western Diet in adolescent depression-current reports. Nutrients.

[17] Ríos-Hernández A, Alda JA, Farran-Codina A, Ferreira-García E, Izquierdo-Pulido M (2017). The Mediterranean Diet and ADHD in children and adolescents. Pediatrics.

[18] Velázquez-López L (2014). Mediterranean-style diet reduces metabolic syndrome components in obese children and adolescents with obesity. BMC Pediatr..

[19] Rahimi H (2020). Dietary approaches to stop hypertension (DASH) score and obesity phenotypes in children and adolescents. Nutr. J..

[20] Mahdavi A (2021). Effects of the dietary approach to stop hypertension (DASH) diet on blood pressure, blood glucose, and lipid profile in adolescents with hemophilia: A randomized clinical trial. Food Sci. Nutr..

[21] Couch SC (2021). Dietary approaches to stop hypertension dietary intervention improves blood pressure and vascular health in youth with elevated blood pressure. Hypertension.

[22] Beigrezaei S (2023). Dietary approaches to stop hypertension (DASH)-style diet in association with gastroesophageal reflux disease in adolescents. BMC Public Health.

[23] Bricarello LP (2021). DASH diet (Dietary Approaches to Stop Hypertension) and overweight/obesity in adolescents: the ERICA study. Clin. Nutr. ESPEN.

[24] Bricarello LP (2020). Association between DASH diet (Dietary Approaches to Stop Hypertension) and hypertension in adolescents: a cross-sectional school-based study. Clin. Nutr. ESPEN.

[25] Corley J (2020). Adherence to the MIND diet is associated with 12-year all-cause mortality in older adults. Public Health Nutr..

[26] Torabynasab K (2023). Adherence to the MIND diet is inversely associated with odds and severity of anxiety disorders: a case–control study. BMC Psychiatry.

[27] Morris MC (2015). MIND diet associated with reduced incidence of Alzheimer’s disease. Alzheimers Dement J. Alzheimers Assoc..

[28] Agarwal P (2018). MIND diet associated with reduced incidence and delayed progression of Parkinsonism in old age. J. Nutr. Health Aging.

[29] Dahl RE (2004). Adolescent brain development: a period of vulnerabilities and opportunities. keynote address. Ann. N. Y Acad. Sci..

[30] Konrad K, Firk C, Uhlhaas PJ (2013). Brain development during adolescence. Dtsch Ärztebl Int..

[31] Prado EL, Dewey KG (2014). Nutrition and brain development in early life. Nutr. Rev..

[32] Asgari E, Chamary M, Bellissimo N, Azadbakht L (2022). Association between adherence to the MIND diet and overweight and obesity in children: An exploratory study. Clin. Nutr. ESPEN.

[33] Krieger N (2001). Theories for social epidemiology in the 21st century: an ecosocial perspective. Int. J. Epidemiol..

[34] Honjo K (2004). Social epidemiology: definition, history, and research examples. Environ. Health Prev. Med..

[35] Thomson JL, Tussing-Humphreys LM, Goodman MH, Landry AS (2019). Diet quality in a nationally representative sample of American children by sociodemographic characteristics. Am. J. Clin. Nutr..

[36] Xu F, Cohen SA, Greaney ML, Hatfield DL, Greene GW (2019). Racial/ethnic disparities in US adolescents’ dietary quality and its modification by weight-related factors and physical activity. Int. J. Environ. Res. Public Health.

[37] Jones L, Ness A, Emmett P (2021). Misreporting of energy intake from food records completed by adolescents: associations with sex, body image, nutrient, and food group intake. Front. Nutr..

[38] Livingstone MBE, Robson PJ, Wallace JMW (2004). Issues in dietary intake assessment of children and adolescents. Br. J. Nutr..

[39] Wallace A, Kirkpatrick SI, Darlington G, Haines J (2018). Accuracy of parental reporting of preschoolers’ dietary intake using an online self-administered 24-h recall. Nutrients.

[40] Barlow SE, and the Expert Committee. (2007). Expert committee recommendations regarding the prevention, assessment, and treatment of child and adolescent overweight and obesity: summary report. Pediatrics.

[41] Centers for Disease Control and Prevention. A SAS Program for the 2000 CDC Growth Charts (ages 0 to <20 years). Published 2019. Accessed November 25, 2020. https://www.cdc.gov/nccdphp/dnpao/growthcharts/resources/sas.htm.

[42] Heeringa S., Berglund P. A guide for population-based analysis of the Adolescent Brain Cognitive Development (ABCD) study baseline data. *bioRxiv*. Published online February 2020:2020.02.10.942011. 10.1101/2020.02.10.942011.

[43] Mercado D. L. et al. Undocumented Latino Immigrants and the Latino Health Paradox. *Am. J. Prev. Med*. 0. 10.1016/j.amepre.2023.02.010 (2023). 10.1016/j.amepre.2023.02.010 PMC10363195 36890084

[44] Sorond FA (2022). Mediterranean diet and brain health in hispanic or latino adults. JAMA Netw. Open.

[45] Antecol H, Bedard K (2006). Unhealthy assimilation: why do immigrants converge to American health status levels?. Demography.

[46] Xie B, Gilliland FD, Li YF, Rockett HRH (2003). Effects of ethnicity, family income, and education on dietary intake among adolescents. Prev. Med.

[47] Gu X, Tucker KL (2017). Dietary quality of the US child and adolescent population: trends from 1999 to 2012 and associations with the use of federal nutrition assistance programs12. Am. J. Clin. Nutr..

[48] Tao MH, Liu JL, Nguyen USDT (2022). Trends in diet quality by race/ethnicity among adults in the United States for 2011–2018. Nutrients.

[49] Shan Z (2019). Trends in dietary carbohydrate, protein, and fat intake and diet quality among US adults, 1999-2016. JAMA.

[50] Rehm CD, Peñalvo JL, Afshin A, Mozaffarian D (2016). Dietary intakes among US adults, 1999-2012. JAMA.

[51] Liu J, Micha R, Li Y, Mozaffarian D (2021). Trends in food sources and diet quality among US children and adults, 2003-2018. JAMA Netw. Open.

[52] Daniel C (2016). Economic constraints on taste formation and the true cost of healthy eating. Soc. Sci. Med..

[53] Hiza HAB, Casavale KO, Guenther PM, Davis CA (2013). Diet quality of Americans differs by age, sex, race/ethnicity, income, and education level. J. Acad. Nutr. Diet..

[54] Regan C, Walltott H, Kjellenberg K, Nyberg G, Helgadóttir B (2022). Investigation of the associations between diet quality and health-related quality of life in a sample of Swedish adolescents. Nutrients.

[55] Solazzo AL (2021). Variation in diet quality across sexual orientation in a cohort of U.S. women. Cancer Causes Control.

[56] Prestemon CE, Grummon AH, Rummo PE, Taillie LS (2022). Differences in dietary quality by sexual orientation and sex in the United States: NHANES 2011-2016. J. Acad. Nutr. Diet..

[57] VanKim NA (2019). Gender expression and sexual orientation differences in diet quality and eating habits from adolescence to young adulthood. J. Acad. Nutr. Diet..

[58] Gower AL, Rider GN, McMorris BJ, Eisenberg ME (2018). Bullying victimization among LGBTQ youth: current and future directions. Curr. Sex. Health Rep..

[59] Watson RJ, Adjei J, Saewyc E, Homma Y, Goodenow C (2017). Trends and disparities in disordered eating among heterosexual and sexual minority adolescents. Int. J. Eat. Disord..

[60] Patterson JG, Jabson JM (2018). Sexual orientation measurement and chronic disease disparities: National Health and Nutrition Examination Survey, 2009–2014. Ann. Epidemiol..

[61] Nelson M, Black AE, Morris JA, Cole TJ (1989). Between- and within-subject variation in nutrient intake from infancy to old age: estimating the number of days required to rank dietary intakes with desired precision. Am. J. Clin. Nutr..

[62] Fuligni GL, Gonzalez CJ, Figueroa R (2022). Adolescents’ proxy reports on obesity-related parenting practices: factorial validity and reliability across four behavioral domains. BMC Public Health.

[63] Walker JL, Ardouin S, Burrows T (2018). The validity of dietary assessment methods to accurately measure energy intake in children and adolescents who are overweight or obese: a systematic review. Eur. J. Clin. Nutr..

[64] American Heart Association. Life’s Essential 8. Accessed June 13, 2023. https://www.heart.org/en/healthy-living/healthy-lifestyle/lifes-essential-8.

[65] Auld M. E. et al. Health Literacy and Health Education in Schools: Collaboration for Action. *NAM Perspect*. 2020:10.31478/202007b. 10.31478/202007b (2020). 10.31478/202007b PMC8916818 35291735

[66] Calfas KJ, Zabinski MF, Rupp J (2000). Practical nutrition assessment in primary care settings: a review. Am. J. Prev. Med.

[67] Daniels SR, Hassink SG, COMMITTEE ON NUTRITION. (2015). The role of the pediatrician in primary prevention of obesity. Pediatrics.

[68] Lane WG, Dubowitz H, Feigelman S, Poole G (2014). The effectiveness of food insecurity screening in pediatric primary care. Int J. Child Health Nutr..

[69] Downer S, Berkowitz SA, Harlan TS, Olstad DL, Mozaffarian D (2020). Food is medicine: actions to integrate food and nutrition into healthcare. BMJ.

[70] Savage JS, Fisher JO, Birch LL (2007). Parental influence on eating behavior: conception to adolescence. J. Law Med. Ethics J. Am. Soc. Law Med. Ethics.

