# Supplementary material for: Social epidemiology of the Mediterranean-dietary approaches to stop hypertension intervention for neurodegenerative delay (MIND) diet among early adolescents: the Adolescent Brain Cognitive Development Study
Source: Pediatr Res. 2023 Dec 15;96(1):230–6. doi: 10.1038/s41390-023-02959-7 (PMC11178675; doi:10.1038/s41390-023-02959-7)
Supplement: Supplementary file 1 — Appendix [file 41390_2023_2959_MOESM1_ESM.docx]

| Appendix A. Comparison of participants included vs excluded | | | |
| --- | --- | --- | --- |
| Sociodemographic characteristics | Included (n= 8,333) | Excluded (n= 2,892) | p |
| Sex (%) |  |  | 0.656 |
| Female | 48.9% | 48.5% |  |
| Male | 51.0% | 51.5% |  |
| Sexual minority (%) |  |  | 0.141 |
| No | 87.4% | 87.6% |  |
| Yes | 4.5% | 4.0% |  |
| Maybe | 3.9% | 3.4% |  |
| Don't understand the question | 3.1% | 3.3% |  |
| Decline to answer | 1.1% | 1.8% |  |
| Race/ethnicity (%) |  |  | <0.001 |
| White | 56.7% | 43.1% |  |
| Latino / Hispanic | 19.3% | 21.8% |  |
| Black | 14.4% | 23.8% |  |
| Asian | 5.3% | 5.9% |  |
| Native American | 2.9% | 3.7% |  |
| Other | 1.3% | 1.8% |  |
| Household income (%) |  |  | <0.001 |
| $24,999 or less | 15.4% | 21.1% |  |
| $25,000 to $49,999 | 18.1% | 20.1% |  |
| $50,000 to $74,999 | 15.7% | 17.1% |  |
| $75,000 to $99,999 | 14.7% | 13.0% |  |
| $100,000 to $199,999 | 26.8% | 21.1% |  |
| $200,000 and greater | 9.4% | 7.7% |  |
| Parent's highest education |  |  | <0.001 |
| College education or more | 85.9% | 81.6% |  |
| High school education or less | 14.1% | 18.4% |  |
| ABCD propensity weights were applied based on the American Community Survey from the US Census. | | | |

| Appendix B. ABCD Study Child Nutrition Assessment: MIND Diet Components: servings and scores | | | |
| --- | --- | --- | --- |
| Diet component | Point awarded for adherence | | "Think about what your child eats in a typical week, during this past year since we last saw you. In a typical week, does your child eat____?" |
|  | 0 | 1 |  |
| Whole grains (servings/day) | <2 | ≥ 3 | Whole grains 3 or more times per day? |
| Green leafy vegetables (servings/week) | <5 | ≥ 6 | Green leafy vegetables 6 or more times per week |
| Other vegetables (servings/day) | 0 | ≥ 1 | Other vegetables 1 or more time per day |
| Berries (servings/week) | <1 | ≥ 2 | Berries 2 or more times per week |
| Red Meat (servings/week) | ≥4 | <3 | Red meats and meat products less than 4 times per week |
| Fish (servings/week) | 0 | ≥ 1 | Fish 1 or more time per week |
| Poultry (servings/week) | <1 | ≥ 2 | Poultry 2 or more times per week |
| Beans (servings/week) | <3 | ≥ 4 | Beans 4 or more times per week |
| Nuts (servings/week) | <3 | ≥ 5 | Nuts 5 or more times per week |
| Fast food (servings/week) | ≥1 | <1 | Fast food or fried food less than 1 time per week |
| Olive Oil (primary) | no | yes | Olive oil is used as the primary oil |
| Butter (servings/day) | ≥1 tbsp | <1 tbsp | Butter or margarine is used less than 1 Tablespoon per day |
| Cheese (servings/week) | ≥1 | <1 | Cheese less than 1 time per week |
| Pastries (servings/week) | ≥5 | <5 | Pastries or sweets less than 5 times per week |
| **Question on wine consumption was ommitted by the ABCD Study. Adolescents were under 21, the legal drinking age in the United States. | | | |
| Reference: Morris MC, Tangney CC, Wang Y, Sacks FM, Barnes LL, Bennett DA, Aggarwal NT. MIND diet slows cognitive decline with aging. Alzheimer's & dementia: the journal of the Alzheimer's Association. 2015; 11(9):1015-1022. | | | |

| Appendix C. Sociodemographic associations with MIND diet sum score in the Adolescent Brain Cognitive Development (ABCD) Study, adjusted for anthropometrics | | |
| --- | --- | --- |
| Independent variables | MIND diet sum score | |
|  | Coefficient (95% CI) | p-value |
| Age | **-0.16 (-0.26, -0.07)** | **0.001** |
| Sex |  |  |
| Female | Reference |  |
| Male | **-0.17 (-0.29, -0.05)** | **0.005** |
| Race/ethnicity |  |  |
| White | Reference |  |
| Asian | **0.38 (0.06, 0.70)** | **0.019** |
| Black | **0.34 (0.12, 0.55)** | **0.002** |
| Latino / Hispanic | **0.74 (0.53, 0.95)** | **<0.001** |
| Native American | 0.35 (-0.01, 0.71) | 0.060 |
| Other | 0.61 (-0.05, 1.3) | 0.072 |
| Sexual minority |  |  |
| No | Reference |  |
| Yes | **-0.64 (-0.94, -0.33)** | **<0.001** |
| Maybe | -0.28 (-0.60, 0.03) | 0.078 |
| Don't understand the question | -0.13 (-0.47, 0.20) | 0.435 |
| Decline to answer | -0.37 (-0.97, 0.23) | 0.22 |
| Household income |  |  |
| $24,999 or less | -0.15 (-0.42, 0.12) | 0.27 |
| $25,000 to $49,999 | **-0.46 (-0.69, -0.22)** | **<0.001** |
| $50,000 to $74,999 | **-0.44 (-0.65, -0.22)** | **<0.001** |
| $75,000 to $99,999 | **-0.37 (-0.56, -0.17)** | **<0.001** |
| $100,000 to $199,999 | **-0.22 (-0.38, -0.06)** | **0.007** |
| $200,000 and greater | Reference |  |
| Parent's highest education |  |  |
| College education or more | Reference |  |
| High school education or less | -0.08 (-0.35, 0.20) | 0.586 |
| Anthropometric factors |  |  |
| BMI-for-age z-score | **0.05 (0.00, 0.11)** | **0.050** |
| Height-for-age z-score | **0.14 (0.05, 0.22)** | **0.001** |
| Weight-for-age z-score | **-0.22 (-0.34, -0.11)** | **<0.001** |
| Bold indicates p<0.05. Models represent the abbreviated output from a linear regression model including adjustment for age, sex, race/ethnicity, sexual orientation, household income, parent education, data collection period, anthropometric factors, and study site. Propensity weights from the Adolescent Brain Cognitive Development Study were applied based on the American Community Survey from the US Census.  *MIND* Mediterranean-DASH (Dietary Approaches to Stop Hypertension) Intervention for Neurodegenerative Delay. | | |

| Appendix D. Adjusted associations of anthropometric measures at Year 1 and MIND (Mediterranean-DASH [Dietary Approaches to Stop Hypertension] Intervention for Neurodegenerative Delay) diet sum score. | | |  |
| --- | --- | --- | --- |
|  |  |  |  |
| Anthropometric measures | B (95% CI) | p-value |  |
| BMI-for-age z-score | **-0.06 (-0.09, -0.02)** | **0.001** |  |
| Height-for-age z-score | 0.02 (-0.03, 0.07) | 0.492 |  |
| Weight-for-age z-score | **-0.09 (-0.14, -0.03)** | **0.002** |  |
| Bold indicates p<0.05. Models adjusts for age, sex, race/ethnicity, sexual orientation, household income, parent education, and study site. Propensity weights from the Adolescent Brain Cognitive Development Study were applied based on the American Community Survey from the US Census. | | |  |
|  |  |  |  |
|  |  |  |  |
